# New insights into β-glucan-enhanced immunity in largemouth bass *Micropterus salmoides* by transcriptome and intestinal microbial composition

**DOI:** 10.3389/fimmu.2022.1086103

**Published:** 2022-12-14

**Authors:** Yuexing Zhang, Mingyu Guo, Ning Li, Zhiyong Dong, Linwei Cai, Bowen Wu, Jianjun Xie, Liang Liu, Lina Ren, Bo Shi

**Affiliations:** ^1^ National Engineering Research Center for Marine Aquaculture, Marine Science and Technology College, Zhejiang Ocean University, Zhoushan, China; ^2^ Kemin AquaScience, Zhuhai, Guangdong, China; ^3^ Zhejiang Marine Fisheries Research Institute, Zhoushan, Zhejiang, China

**Keywords:** largemouth bass, β-Glucan, growth, immunity, *Aeromonas schubertii*

## Abstract

β-glucan is widely used in aquaculture due to its immunostimulatory effects, but the specific effect and potential regulatory mechanism on largemouth bass (*Micropterus salmoides*) are still unclear. Here, we evaluated the effects of β-glucan on growth, resistance to *Aeromonas schubertii*, intestinal health, and transcriptome of largemouth bass to reveal the potential regulators, metabolic pathways, and altered differential microbiota. Four experimental diets were designed with β-glucan supplementation levels of 0 (control), 100 (LA-100), 200 (MA-200), and 300 (HA-300) mg kg^-1^, and each diet was fed to largemouth bass (79.30 ± 0.50 g) in triplicate for 70 days, followed by a 3-day challenge experiment. Results showed that different β-glucan supplementations had no significant effects on growth performance and whole-body composition. Fish fed a diet with 300 mg kg^-1^ β-glucan significantly increased the activity of lysozyme than those fed diets with 0 and 100 mg kg^-1^ β-glucan. In addition, the survival rate of largemouth bass in β-glucan supplementation groups was significantly higher than the control group at 12- and 24-h challenge by *Aeromonas schubertii*. Transcriptome analysis showed that a total of 1,245 genes were differentially expressed [|log_2_(fold change)| ≥1, *q*-value ≤0.05], including 109 immune-related differentially expressed genes (DEGs). Further analysis revealed that significantly upregulated and downregulated DEGs associated with immunity were mapped into 12 and 24 pathways, respectively. Results of intestinal microflora indicated that fish fed a diet with 300 mg kg^-1^ β-glucan had higher bacterial richness and diversity as evaluated by Sobs, Chao, Ace, and Simpson indices, but no significant differences were found in the comparison groups. Furthermore, 300 mg kg^-1^ β-glucan significantly increased the relative abundance of *Mycoplasma* and decreased *Proteobacteria* (mainly *Escherichia-Shigella* and *Escherichia coli*) and *Bacillus anthracis* in largemouth bass intestinal microflora. The findings of this study provided new insights that will be valuable in future studies to elucidate the mechanism of immunity enhancement by β-glucan.

## 1 Introduction

With the development and optimization of compound feed, the farming production and scale of the largemouth bass (*Micropterus salmoides*) have been expanding and now it has become one of the fastest growing cultured fish species in Chinese freshwater aquaculture (1). However, overcrowding and poor water quality due to intensive farming increased the susceptibility of fish to infection or disease (2). *Nocardia seriolae*, *Edwardsiella piscicida*, and *Aeromonas hydrophila* are the serious pathogens causing disease and death of largemouth bass (3). To alleviate disease problems, antibiotics and some drugs have been used in aquaculture, while the overuse of antibiotics will produce antibiotic-resistant bacteria and the residue and accumulation of drug will cause food safety hazards (4). Hence, eco-friendly disease prevention measures need to be found to alleviate the occurrence of disease and promote sustainable culture of fish. A promising alternative to improve the immunity of fish is supplementation with functional feed additives. Immunostimulants are effective additives that activate nonspecific immunity to improve the immune system of organisms. Numerous studies have proposed that delivery of immunostimulants as a dietary supplement in feed can improve immunity of multiple fish species (5). Thus, supplementing immunostimulants in feed is one of the effective ways to alleviate disease problems.

β-glucan has received heightened attention by feed manufacturers as a natural, safe, and economical immunostimulant that can stimulate the immune response of aquatic animals. β-glucan is a polysaccharide extracted from the cell wall of cereals, algae, yeast, or bacteria. Different sources of β-glucan have different structures and thus express different biological activities (6). Currently, most commercially available β-glucans are derived from yeast or cereal, but they are partially water-soluble or insoluble. With the development of extraction technology, microalgae have been considered as a potential source of β-glucans and can produce various β-glucans with different structures and solubilities. However, limited research has been conducted on algae-derived β-glucan in fish.

The immunostimulatory effects of β-glucan have been reported in different fish species including rohu (*Labeo rohita*), rainbow trout (*Oncorhynchus mykiss*), Atlantic salmon (*Salmo salar* L.), red sea bream (*Pagrus major*), koi (*Cyprinus carpio koi*), mirror carp (*Cyprinus carpio* L.), and crustacean (7–13), but it has not been evaluated on largemouth bass. β-glucan can interact with the immune system to enhance the resistance of fish to pathogens. Several studies have reported that β-glucan induced increased resistance of fish to several bacterial pathogens by increasing the levels of complement and lysozyme (LZM), enhancing phagocytic and bactericidal activities of phagocytes (6, 14). In addition, studies reported that β-glucan plays an important role in improving the intestinal environment by promoting beneficial microorganisms, acidifying the intestinal tract, and reducing harmful metabolites in the intestine (6, 13). Obviously, β-glucan has multifaceted regulatory effects on the immune system. Therefore, the overall aim of this study was to investigate the effects of prolonged application of algae-derived β-glucan on growth, immunity, and resistance to *A. schubertii* in largemouth bass and to reveal the potential mechanism by which β-glucan modulates the immune system using Illumina MiSeq 16S rRNA gene and transcriptome sequencing technology.

## 2 Materials and methods

### 2.1 Experimental diets

Four isonitrogenous (~530 g kg^-1^) and isoenergetic (~22 MJ kg^-1^) diets were formulated to contain different levels of β-glucan (**Table S1**). A basal diet was supplemented with 0 (control), 100 (LA-100), 200 (MA-200), and 300 (HA-300) mg kg^-1^ β-glucan (algae-derived β-glucan). The fish meal, poultry by-product meal, soybean meal, and soy protein concentrate were used as main protein sources, and fish oil and soybean oil were used as lipid sources. Experimental diets were processed in Buhler (Changzhou) Machinery Co., Ltd., and the feed processing technology was strictly in accordance with Buhler Aquatic processing scheme. Briefly, the cribble of all ingredients was carried out in a horizontal hammer mill (AHZC-0655), then sent to the vertical shaft micronizer (AHFL-110) for superfine grinding. The premix and superfine grinding ingredients were weighed and mixed in a single shaft paddle mixer (AHML-1000). Before extrusion, the mixed ingredients were preconditioned by conditioner (BCCC-22) to be matured in a humid and hot environment, then extruded by a twin-screw extruder (BCCG-62). The pellets were sucked into the dryer (BDBDP2G0.5C) for dying until the moisture is around 8%. The oil was vacuum-sprayed at the Feed Technology Laboratory of the Sino-European Aquatic Nutrition and Feed Resources Institute, Zhejiang Ocean University (SEANUTR-ZJOU). The oil mixture (the mix of fish oil and soybean oil was 1:1) was preheated to 50°C, then vacuum-sprayed in a vertical vacuum coating machine (ZJB-100). The pellets were quiesce for 24 h and sieved, damaged pellets were removed, and the remaining pellets were stored at -20°C until use.

### 2.2 Fish feeding and experimental conditions

Juvenile largemouth bass (~5 g) were obtained from a local hatchery (Hongli Aquaculture Co., Huzhou, Zhejiang) and reared in 22 m^2^ fiberglass breeding pool to acclimate the laboratory conditions with commercial feed (~520 g kg^-1^ protein, ~80 g kg^-1^ lipid). The 70-day feeding trial was conducted in SEANUTR-ZJOU. A total of 600 juveniles (79.30 ± 0.50 g) were randomly assigned to 12 cylindrical fiberglass tanks (1,000 L) in recirculated aquaculture system, and each diet was assigned to three replicates with 50 fish per tank. Daily management procedure of the 70-day feeding trial followed that of a previous study (15). Briefly, largemouth bass were manually fed three times per day at 8:00 a.m., 2:00 p.m., 8:00 p.m.; all uneaten pellets were immediately siphoned out and quantified by the method of Zhang et al. (16). Tentative daily biomass of 10% was determined based on the average feed intake over the past 3 days, with more feed given at the end of each meal if fish showed signs of feeding. Each tank was supplied with seawater at a flow rate of 4–5 L min^-1^, and water quality parameters were measured daily including ammonia nitrogen content <0.25 mg L^-1^, nitrite nitrogen <0.5 mg L^-1^, pH 7.0–7.5, dissolved oxygen of 5.0 ± 0.3 mg L^-1^, and temperature 26°CC–28°CC.

### 2.3 *Aeromonas schubertii* challenge experiment

*A. schubertii* was isolated from diseased largemouth bass and cultured at 28°CC for 24 h, centrifuged at 10,000 × g for 10 min at 4°CC, and resuspended in 1 × PBS. After the feeding experiment, 25 fish per tank were fed as before and recovered from weighing and sampling stress by 2-week acclimation. Then, 300 largemouth bass (~300 g) were intraperitoneally injected with 150 µl *A. schubertii* suspension (3 × 10^9^ CFU ml^-1^) (3), while 150 µl sterile saline solution (0.85%) was also injected as the blank control group. The survival rate of largemouth bass was recorded every 12 h (0, 12, 24, 48, and 72 h) without any diet. No mortality was found in the blank control group, suggesting that no fish died because of injection stress.

### 2.4 Sampling

At the termination of the feeding experiment, fish were fasted for 24 h and anesthetized with MS-222. All fish were counted and weighed individually to assess the growth index [weight gain rate (WGR), specific growth rate (SGR), feed intake (FI), feed conversion ratio (FCR)]. Morphologic index including condition factor (CF), gonadosomatic index (GSI), hepatosomatic index (HSI), and viscerosomatic index (VSI) were calculated by measuring the body length and weight of the whole body, liver, gonad, and viscus from five fish per tank. Five fish from each tank were collected to analyze the whole body composition. Blood samples were collected from a further five fish per tank and centrifuged at 3,000 × g for 10 min at 4°CC, frozen in liquid N_2_, then kept at -80°CC until analysis of serum biochemical parameters. Liver was collected from five fish per tank and immediately immersed in RNA keeper (Vazyme, China), prestored at 4°C for 24 h, and then transferred to -80°C until transcriptome sequencing. The hindgut of five fish from each tank was removed aseptically, collected into sterile tubes, rapidly frozen in liquid N_2_, and then kept in -80°CC for intestinal microflora analyses.

### 2.5 Proximate compositions and hematological parameters

Pretreatment of the whole-body sample was in reference to a previous study (15). Briefly, samples were pooled per tank and homogenized by a meat grinder, dried at 120°CC for 30 min, rehomogenized in the high-speed tissue homogenizer, then dried in 75°CC oven, and finely ground into powder before analysis. Dry matter (105°C to constant weight), crude protein (Kjeldahl N, Opsis KD-310, Sweden), crude lipid (HCl hydrolysis and ether extraction, Opsis SX110A and SX-360, Sweden), ash (550°C, Muffle furnace), and gross energy (Parr, 1271, USA) in diets and whole body were analyzed by the standard methods of the Association of Official Analytical Chemists (AOAC) (17).

Hematological parameters including superoxide dismutase (SOD), catalase (CAT), and LZM were determined by the commercial kits (Nanjing Jiancheng Bio Inst, Nanjing, China) and performed according to the manufacturer’s instruction.

### 2.6 Transcriptional analysis

#### 2.6.1 RNA extraction and library construction

The livers obtained from the control, LA-100, MA-200, and HA-300 groups were entrusted to BGI-Wuhan Technology Service Co., Ltd., for RNA extraction, quality control, library construction, and RNA sequencing. Total RNA was extracted from the liver using TRIzol Reagent (Invitrogen, CA, USA) according to the manufacturer’s protocol. Subsequently, the concentration and quality of RNA were assessed by ND 2000 (Thermo Fisher Scientific, USA) and Agilent 2100 bioanalyzer (Thermo Fisher Scientific, MA, USA). The cDNA fragments were amplified by PCR, and products were purified by Ampure XP Beads. Library quality was validated on the Agilent 2100 bioanalyzer. High-quality RNA samples were used for library preparation and performed on an Illumina HiSeq4000 sequencer according to the manufacturer’s specifications (Illumina).

#### 2.6.2 Data analysis

All raw reads (accession number: SRR21783676, SRR21783677, SRR21783678, SRR21783679, SRR21783680, SRR21783681) were filtered with SOAPnuke software; afterward, clean reads were stored in FASTQ format. The HISAT2 and Bowtie2 software were used to align clean reads to the reference genome (GCF_014851395.1_ASM1485139v1_NCBI) and coding gene set (18, 19). Gene expression levels for each sample were calculated using RSEM software and normalized into fragment per kilobase of transcript per million base pairs sequenced (FPKM) (19, 20). The functional annotation and classification of largemouth bass transcriptome were shown in **Figure S2**. Differentially expressed genes (DEGs) were screened between two comparison groups (control *vs*. HA-300) using the DEGSeq2, with |log_2_(fold change)| ≥1 and *q*-value ≤0.05 (21, 22). Gene Ontology (GO) and Kyoto Encyclopedia of Gene and Genomes (KEGG) enrichment analysis of annotated DEGs were performed by Phyper based on the hypergeometric test, with *q*-value ≤0.05 being considered as significantly enriched.

### 2.7 Intestinal microbial analysis

#### 2.7.1 Intestinal DNA extraction, PCR amplification, and illumina miSeq sequencing

DNA was extracted from the hindgut of five largemouth bass at equal concentrations in each sample. The bacterial community DNA was performed according to the instructions of EZNA^®^ soil DNA kit (Omega Bio-tek, Norcross, GA, USA). The concentration and quality of DNA were verified using ND 2000 and 1% agarose gel electrophoresis. Amplification of the 16S rRNA gene was performed with primer pairs (338F: 5’-ACTCCTACGGGAGGCAGCAG-3’ and 806R: 5’-GGACTACHVGGGTWTCTAAT-3’) by an ABI GeneAmp^®^ 9700 PCR thermocycler (ABI, CA, USA). The PCR amplification was performed with a 20 μl reaction volume containing 4 μl of 5× TransStart FastPfu buffer, 0.8 μl (each) of forward and reverse primers (5 μM), 2.0 μl dNTPs (2.5 mM), 0.4 μl TransStart FastPfu DNA polymerase, 10 ng template DNA, and ddH_2_O up to 20 μl. The PCR program was 95°C for 3 min, followed by 27 cycles of 95°C for 30 s, 55°C for 30 s, 72°C for 30 s, and then 72°C for 10 min. PCR products were recovered using 2% agarose gel, purified using the AxyPrep DNA Gel Extraction Kit (Axygen Biosciences, Union City, CA, USA), and quantified using Quantus™ Fluorometer (Promega, USA) for the recovered products. Library construction and sequencing were performed using NEXTflexTM Rapid DNA-Seq Kit (Bioo Scientific, USA) and Illumina’s MiSeq PE300/NovaSeq PE250 platform (Shanghai Meiji Biomedical Technology Co., Ltd.). All raw data were deposited into the NCBI SRA database (accession number: SRR21783450, SRR21783451, SRR21783452, SRR21783453, SRR21783454, SRR21783455).

#### 2.7.2 Bioinformatic analysis

The raw reads were demultiplexed, quality-filtered by FASTP and merged by FLASH (23). Operational taxonomic unit (OTU) clustering of sequences (based on 97% similarity) and removal of chimeras were performed using UPARSE software (24). The taxonomy of each OTU representative sequence was analyzed by RDP Classifier against the 16S rRNA database using confidence threshold of 0.7 (25). Taxonomic richness and diversity estimators including observed richness (Sobs), Chao1 estimator (Chao), ACE estimator (Ace), Shannon diversity index (Shannon), Simpson diversity index (Simpson), and Good’s coverage (Coverage) were determined using the Mothur software. The relative abundance of taxa for each sample was generated into domain, kingdom, phylum, class, order, family, genus, and species levels. The Linear discriminant analysis Effect Size (LEfSe) was determined using the LEfSe software to reflect communities or species that produced significant differential effects, with linear discriminant analysis (LDA) score >2 and Wilcoxon rank-sum test (*P* < 0.05) being used for significant difference analysis.

### 2.8 Statistical analysis

Statistical analysis was conducted using the SPSS 20 software (IBM SPSS Statistics 20). Results are presented as means and pooled SEM of three replicates (n = 3). All data were checked for normality and homogeneity of variances and were normalized when appropriate. Results were analyzed by one-way ANOVA to investigate differences among treatments followed by Duncan’s multiple range test, with *P* < 0.05 being considered as a significantly different level. The calculations were listed in the **Supplementary Materials**.

## 3 Results

### 3.1 Growth performance and body composition

As shown in **Tables 1**, **2**, β-glucan supplementation did not result in a significant difference in growth performance (FI, WG, FCR, and SGR), morphologic index (HSI, VSI, GSI, and CF), body composition (moisture, protein, fat, ash, gross energy), protein retention efficiency, and energy retention efficiency in largemouth bass (*P* > 0.05).

**Table 1:** Growth performance and morphologic index of largemouth bass fed diets with different levels of β-glucan.

Items	Diet				*P*-value	PooledSEM^1^
	Control	LA-100	MA-200	HA-300		
FBW, g fish^-1^	295	295	295	292	0.98	10.2
FI, g DM fish^-1^	190	193	191	188	0.83	7.05
WGR, %	272	271	273	268	0.98	14.3
FCR, g FI (g WG)^-1^	0.89	0.90	0.89	0.88	0.49	0.01
SGR, % d^-1^	1.90	1.90	1.90	1.89	0.98	0.06
HSI, %	1.71	1.76	1.61	1.51	0.13	0.14
VSI, %	8.74	8.74	8.35	8.75	0.59	0.48
GSI, %	1.54	1.83	0.93	1.34	0.38	0.69
CF, g cm^-3^	2.98	3.12	3.06	2.98	0.14	0.08

^1^ Pooled standard error of means.

Values are means and pooled SEM (n = 3); different superscript letters indicate significant differences among treatments (P < 0.05).

CF, condition factor; FBW, final body weight; FCR, feed conversion ratio; FI, feed intake; GSI, gonadosomatic index; HSI, hepatosomatic index; SGR, specific growth rate; VSI, viscerosomatic index; WGR, weight gain rate.

**Table 2:** Whole body composition and nutrient retention efficiency of largemouth bass fed diets with different levels of β-glucan.

Items	Diet				*P*-value	PooledSEM	
	Control	LA-100	MA-200	HA-300		
Moisture, g kg^-1^	661	657	659	660	0.32	3.65
Crude protein, g kg^-1^	176	177	177	178	0.18	1.26
Crude fat, g kg^-1^	123	126	125	121	0.39	4.02
Ash, g kg^-1^	36.2	36.1	36.5	35.9	0.89	1.12
Gross energy, MJ kg^-1^	8.79	8.97	8.87	8.80	0.41	0.16
Protein retention efficiency, %	38.0	38.4	38.4	38.4	0.77	0.69
Energy retention efficiency, %	47.6	47.9	47.6	47.9	0.98	1.29

Values are means and pooled SEM (n = 3); different superscript letters indicate significant differences among treatments (P < 0.05).

### 3.2 Serum biochemical parameters

Effects of different levels of β-glucan supplementation on activities of enzymes related to immunity and oxidation resistance in largemouth bass serum are presented in **Table 3**. Fish fed a diet with 300 mg kg^-1^ β-glucan significantly had increased activity of LZM compared to those fed diets with 0 and 100 mg kg^-1^ β-glucan (*P <* 0.05), while no differences were found in activities of SOD and CAT in largemouth bass serum (*P* > 0.05).

**Table 3:** Serum biochemical parameters of largemouth bass fed diets with different levels of β-glucan.

Items	Diet				*P*-value	PooledSEM
	Control	LA-100	MA-200	HA-300		
SOD, U ml^-1^	21.5	20.1	22.4	20.3	0.34	1.86
CAT, U ml^-1^	3.22	2.72	2.61	2.93	0.88	1.08
LZM, μg ml^-1^	83.3^b^	74.3^b^	92.2^ab^	105^a^	0.03	13.3

Values are means and pooled SEM (n = 3); different superscript letters indicate significant differences among treatments (P < 0.05).

CAT, catalase; LZM, lysozyme; SOD, superoxide dismutase.

### 3.3 Survival rate of largemouth bass after *Aeromonas schubertii* challenge

**Figure 1:**
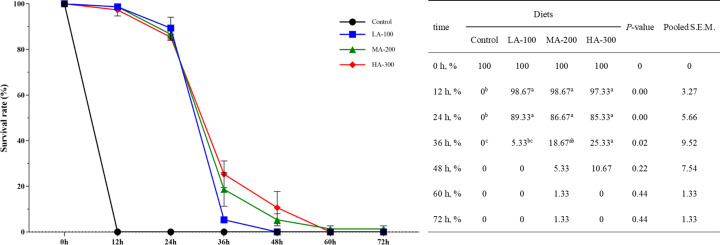
Survival rate of largemouth bass within 72 h after the challenge with *Aeromonas schubertii*. Values are means and pooled SEM (n = 3); different superscript letters indicate significant differences among treatments (*P* < 0.05).

As shown in **Figure 1**, the survival rate of largemouth bass in β-glucan supplementation groups (LA-100, MA-200, and HA-300) was significantly higher than that of the control group at 12 and 24 h (*P <* 0.05). The highest survival rate of largemouth bass after *A. schubertii* challenge was found in fish fed a diet containing 300 mg kg^-1^ β-glucan than those fed diets with 0 and 100 mg kg^-1^ β-glucan at 36 h (*P <* 0.05). Notably, all fish fed a diet without β-glucan supplementation died after the 12-h challenge, but fish in the β-glucan supplementation groups survived after the 72-h challenge, suggesting that β-glucan could improve the resistance of largemouth basses to *A. schubertii*.

### 3.4 Transcriptional analysis of largemouth bass liver

#### 3.4.1 Sequencing and mapping

As shown in **Table 4**, a total of six cDNA libraries including three control libraries (control-1, control-2, control-3) and three HA-300 libraries (HA-300-1, HA-300-2, HA-300-3) with 43.8 million raw reads were constructed. After filtration, the clean reads range from 42.8 to 43.0 million (clean read ratio is about 98%). The percentages of Q20 and Q30 were above 98.0% and 94.3%, indicating that the quality of all samples was qualified and could be used for subsequent data analysis. The sequence length of all unigenes is shown in **Figure S1**, and the length of most transcripts is longer than 3,000 nt.

**Table 4:** Summary of transcriptome sequencing and mapping for largemouth bass.

Group	Sample	Raw reads (10^6^)	Clean reads (10^6^)	Clean bases (Gb)	Q20 (%)	Q30 (%)	Clean reads ratio (%)	Total mapping (%)
Control	Control-1	43.8	43.0	6.45	98.2	94.8	98.1	95.9
	Control-2	43.8	42.9	6.43	98.1	94.4	97.8	95.3
	Control-3	43.8	43.0	6.45	98.1	94.4	98.1	95.7
HA-300	HA-300-1	43.8	42.8	6.43	98.1	94.6	97.8	95.1
	HA-300-2	43.8	43.0	6.45	98.0	94.3	98.1	95.4
	HA-300-3	43.8	43.0	6.46	98.1	94.5	98.2	95.4

#### 3.4.2 Identification of differentially expressed genes (DEGs)

**Figure 2:**
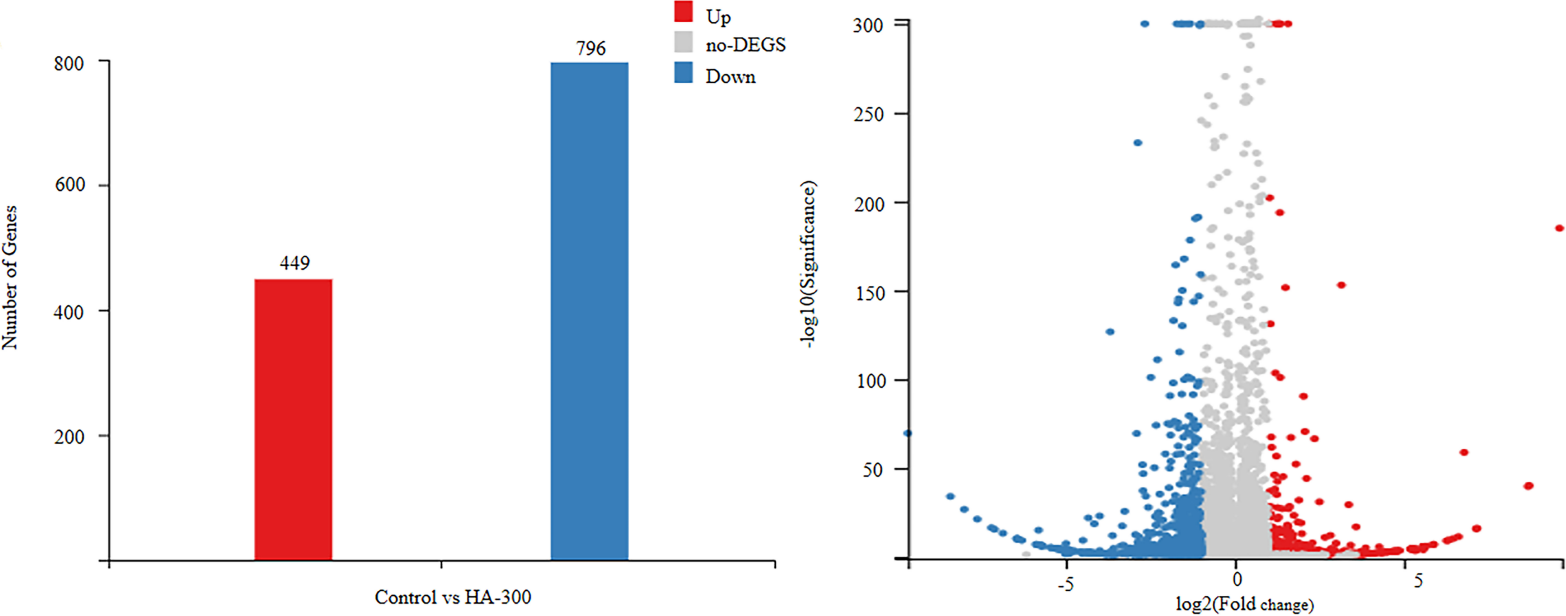
Differentially expressed genes (DEGs) |log_2_(fold change)| ≥1, *q*-value ≤0.05] in the liver transcriptome of largemouth bass fed diets with 0 and 300 mg kg^-1^ β-glucan. The blue dots and column represent significantly downregulated DEGs, and the red blue dots and column represent significantly upregulated DEGs, the gray signifies no DEGs.

The transcriptome analysis was performed between comparison groups (control *vs*. HA-300) to identify DEGs [|log_2_(fold change)| ≥1 and *q*-value ≤0.05] in response to different levels of β-glucan supplementation. Specifically, a total of 1,245 DEGs were obtained; fish fed a diet with 300 mg kg^-1^ β-glucan showed 449 significantly upregulated DEGs and 796 significantly downregulated DEGs compared with the control group (**Figure 2**).

#### 3.4.3 GO annotations and KEGG classification of DEGs

**Figure 3:**
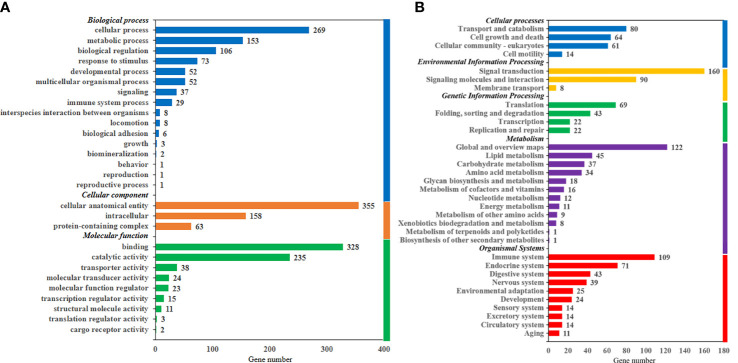
Gene Ontology annotations **(A)** and Kyoto Encyclopedia of Gene and Genomes classification **(B)** of differentially expressed genes in the liver transcriptome of largemouth bass.

As shown in **Figure 3**, DEGs were divided into three categories, including biological process (38.96%), cellular component (28.02%), and molecular function (33.02%). According to KEGG terms, all DEGs were classified into five categories, including organismal systems (27.77%), metabolism (23.95%), environmental information processing (19.68%), cellular processes (16.70%), and genetic information processing (11.90%). GO and KEGG classifications of all unigenes in the live transcriptome of largemouth bass are shown in **Figure S2**.

#### 3.4.4 KEGG enrichment analysis of immune-related DEGs

To further investigate the effect of β-glucan on the immunity of largemouth bass, KEGG enrichment analysis was performed (*q*-value <0.05, **Table 5**). The significantly upregulated DEGs associated with immunity were mapped to 12 pathways, including chemokine signaling pathway, NOD-like receptor signaling pathway, complement and coagulation cascades, interleukin (IL)-17 signaling pathway, and NF-kappa B signaling pathway (top 5 pathways). Accordingly, significantly downregulated DEGs associated with immunity were mapped to 24 pathways, including intestinal immune network for IgA production, cytosolic DNA-sensing pathway, C-type lectin receptor signaling pathway, NOD-like receptor signaling pathway, and NF-kappa B signaling pathway (top 5 pathway). The 109 immune-related DEGs were summarized in **Table S2**.

**Table 5:** The significantly enriched immune-related pathways and corresponding DEGs in the liver transcriptome of largemouth bass (*q*-value <0.05).

Pathway ID	Pathway name	*q*-value	Rich ratio	DEGs in the corresponding pathway^1^
***Upregulated***				
Ko04062	Chemokine signaling pathway	2.80e-5	0.03	*xcr1*, *pak1*, *cxcr1*, *ptk2b*-like, *il8*-like, *ccl7*, *ccl5*, permeability factor 2-like
Ko04621	NOD-like receptor signaling pathway	1.87e-4	0.02	*nlrc3, trmp2-like, il8, nlrp12, ccl7*, *vdac2*-like, *lrrc39*, permeability factor 2-like
Ko04610	Complement and coagulation cascades	4.01e-4	0.03	*cfh*-like, urokinase plasminogen activator surface receptor-like, *f10*-like, B2 bradykinin receptor-like
Ko04657	IL-17 signaling pathway	4.01e-4	0.03	*mmp18*-like, *il8*-like, *ccl7*, protein S100-B-like, *kiaa1522*
Ko04064	NF-kappa B signaling pathway	8.11e-4	0.03	*il8*-like, *trim110*, *ccl5*, permeability factor 2-like
Ko04670	Leukocyte transendothelial migration	9.79e-4	0.02	*ptk2b*-like, *cldn11a*
Ko04622	RIG-I-like receptor signaling pathway	1.22e-3	0.04	*cyld*-like, *il8*-like, *trim110*, permeability factor 2-like
Ko04060	Cytokine-cytokine receptor interaction	2.03e-3	0.02	chemokine XC receptor 1-like, *cxcr1*-like, *il8*-like, *ccl5*, permeability factor 2-like
Ko04620	Toll-like receptor signaling pathway	3.37e-3	0.03	*il8*-like, *map2k7*, *ccl5*, permeability factor 2-like
Ko04072	Phospholipase D signaling pathway	0.01	0.02	*cxcr1*-like, *ptk2b*-like, *kitb*, permeability factor 2-like
Ko04672	Intestinal immune network for IgA production	0.04	0.02	*mpz*-like, *icosl*-like, *clm1*
Ko04668	TNF signaling pathway	0.04	0.02	*ccl7*, protein jagged-1a-like, *map2k7*
***Downregulated***				
Ko04672	Intestinal immune network for IgA production	1.31e-8	0.06	*plgr*-like, nectin-4-like, *tnfrsf13b*-like, *vtcn1*-like, *il10*
Ko04623	Cytosolic DNA-sensing pathway	5.71e-8	0.09	*tnip1*-like, *il1β*, *rpac1*-like, *polr3d*, *polr3c*, *polr2h*
Ko04625	C-type lectin receptor signaling pathway	1.74e-5	0.04	*tnip1*-like, *septin2*-like, *il1β*, *egr2b*, proto-oncogene tyrosine-protein kinase Src-like, *lyg*-like, *rhes*-like, *il10*
Ko04621	NOD-like receptor signaling pathway	3.45e-4	0.02	*tnip1*-like, *septin2*-like, *syngr3a*, *il1β*, *lyg*-like, *syngr1*-like
Ko04064	NF-kappa B signaling pathway	7.35e-4	0.04	*tnip1*-like, *syngr3a*, *il1β*, *syngr1*-like
Ko04650	Natural killer cell mediated cytotoxicity	8.39e-4	0.03	*syngr1*-like, *rhes*-like, *tnfrsf10a*-like
Ko04662	B cell receptor signaling pathway	1.29e-3	0.03	*tnip1*-like, protein FAM110A-like, low-affinity immunoglobulin gamma Fc region receptor II-c-like, *rhes*-like
Ko04666	Fc gamma R-mediated phagocytosis	1.40e-3	0.03	low-affinity immunoglobulin gamma Fc region receptor II-c-like, *marcksl1b*, phospholipid phosphatase 1-like
Ko04640	Hematopoietic cell lineage	1.42e-3	0.03	*fam110a*-like, *il1β*, *tfr1b*
Ko04664	Fc epsilon RI signaling pathway	2.02e-3	0.04	*rhes*-like, *ncoa7*-like
Ko04620	Toll-like receptor signaling pathway	2.07e-3	0.03	*tnip1*-like, *syngr3a*, *il1β*, *lyg*-like, *syngr1*-like, *tlr9*
Ko03020	RNA polymerase	2.39e-3	0.04	*rpac1*-like, *polr3d*, *polr3c*, *polr2h*
Ko04062	Chemokine signaling pathway	2.39e-3	0.02	*tnip1*-like, *arrb1*, *arr3b*, *il8*, proto-oncogene tyrosine-protein kinase Src-like, *rhes*-like, *ttc27*-like
Ko04072	Phospholipase D signaling pathway	0.01	0.02	*rhes*-like, phospholipid phosphatase 1-like, *thada*
Ko04145	Phagosome	0.01	0.02	low-affinity immunoglobulin gamma Fc region receptor II-c-like, *tfr1b*, *ctsl.1*
Ko04010	MAPK signaling pathway	0.01	0.01	*arrb1*, *arr3b*, *syngr3a*, *il1β*, *syngr1*-like, *rhes*-like, *hspa1l*, *ttc27*-like
Ko04670	Leukocyte transendothelial migration	0.02	0.02	*myl7*, *cldn5*-like
Ko04668	TNF signaling pathway	0.02	0.02	*tnip1*-like, *syngr3a*, *il1β*, *lyg*-like, *syngr1*-like
Ko04217	Necroptosis	0.03	0.02	*septin2*-like, *il1β*, *lyg*-like, *tnfrsf10a*-like,
Ko04612	Antigen processing and presentation	0.03	0.03	protein disulfide-isomerase A3-like, *hspa4a*, *hspa1l*, *ctsl.1*
Ko04060	Cytokine-cytokine receptor interaction	0.03	0.02	*il1β*, *il8*, *tnfrsf13b*-like, *il12rb2l*, *il10*, *tnfrsf10a*-like
Ko04340	Hedgehog signaling pathway	0.03	0.04	*arr3b, arrb1*, *ttc27*-like
Ko04380	Osteoclast differentiation	0.03	0.02	*tnip1*-like, *syngr3a*, *il1β*, low-affinity immunoglobulin gamma Fc region receptor II-c-like, *syngr1*-like
Ko04610	Complement and coagulation cascades	0.03	0.02	*f13a*-like, *thada*, *atⅢ*-like

^1^ Abbreviations for **Table 5** were listed in **Table S2** of the **Supplementary Material**.

### 3.5 Intestinal microbial analysis

#### 3.5.1 Intestinal microflora structure at the phylum and class levels

**Figure 4:**
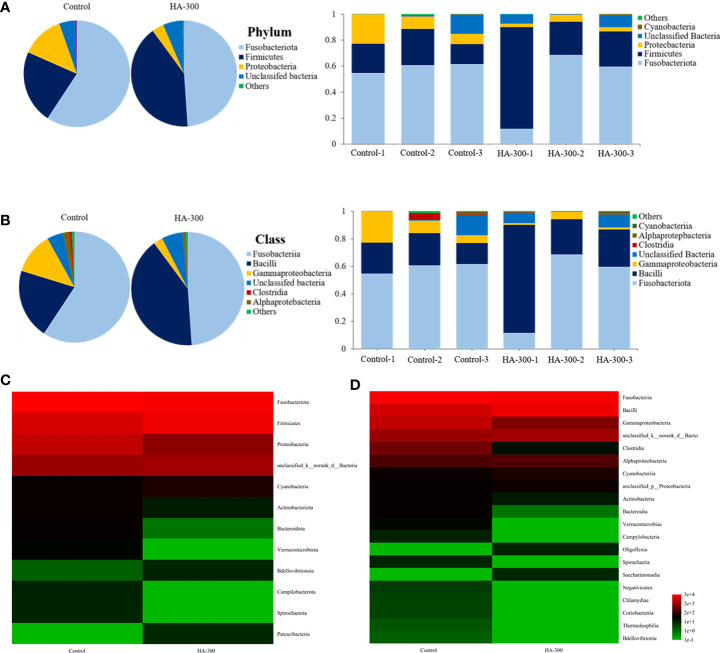
Comparisons of the intestinal microflora structure at the phylum and class levels of largemouth bass (n = 3). **(A)** Percentage distribution and relative abundance of intestinal microflora at the phylum level. **(B)** Percentage distribution and relative abundance of intestinal microflora at the class level. **(C, D)** Heatmap of intestinal microflora abundance at the phylum and class levels.

As shown in **Figure 4A**, the dominant bacteria at the phylum level were *Fusobacteria*, *Firmicutes*, and *Proteobacteria*. Specifically, *Fusobacteria*, *Firmicutes*, and *Proteobacteria* were 59.29%, 22.30%, 13.03% in the control group and 48.92%, 41.00%, and 3.64% in the HA-300 group, respectively. The dominant bacteria of largemouth bass at the class level were *Fusobacteria*, *Bacilli*, and *Gammaproteobacteria* (**Figure 4B**). Specifically, *Fusobacteria*, *Bacilli*, and *Gammaproteobacteria* were 9.30%, 22.44%, and 12.18% in the control group and 48.92%, 40.92%, 2.64% in the HA-300 group, respectively. Heatmaps were used to further compare the relative abundance of intestinal microflora between the control and HA-300 groups at the phylum (**Figure 4C**) and class (**Figure 4D**) levels. *Fusobacteriota*, *Proteobacteria*, *Actinobacteriota*, *Bacteroidota*, and *Verrucomicrobiota* were more abundant in the control group, and *Firmicutes* and *Cyanobacteria* were more abundant in the HA-300 group at the phylum level. *Fusobacteriia*, *Gammaproteobacteria*, *Clostridia*, *Bacteroidia*, and *Verrucomicrobiae* were more abundant in the control group, and *Bacilli* was more abundant in the HA-300 group at the class level.

#### 3.5.2 Alpha diversity analysis

**Figure 5:**
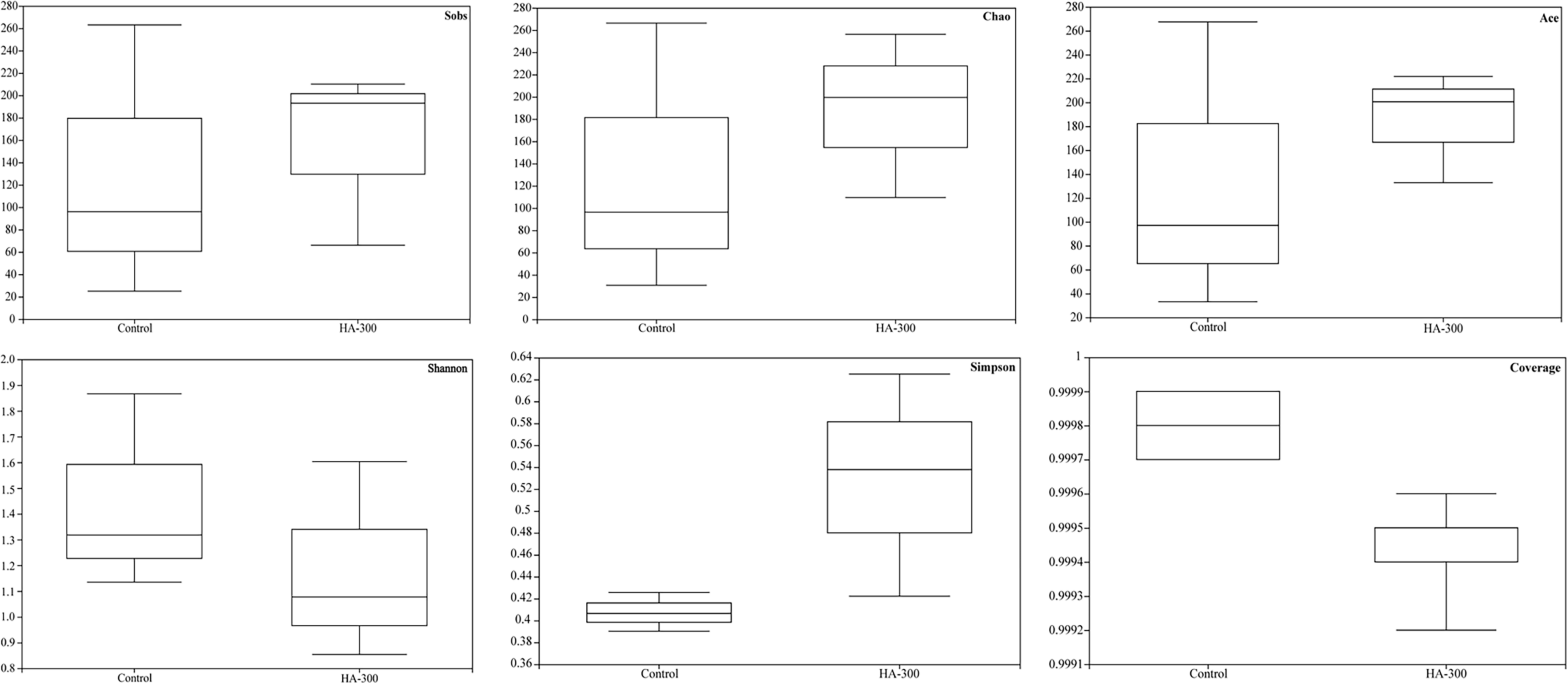
Boxplot for evaluating diversity and richness of intestinal microflora of largemouth bass based on the Sobs, Chao, Ace, Shannon, Simpson, and Coverage indices (n = 3).

Sobs, Ace, and Chao reflect community richness, and Shannon and Simpson represent community diversity, with Coverage being used to evaluate community coverage. No significant difference was found in the alpha diversity analysis including Sobs, Chao, Ace, Shannon, Simpson, and Coverage indices between the control and HA-300 groups (**Figure 5**). However, fish fed a diet with 300 mg kg^-1^ β-glucan showed higher values in the Sobs, Chao, Ace, and Simpson indices.

#### 3.5.3 LEfSe analysis

**Figure 6:**
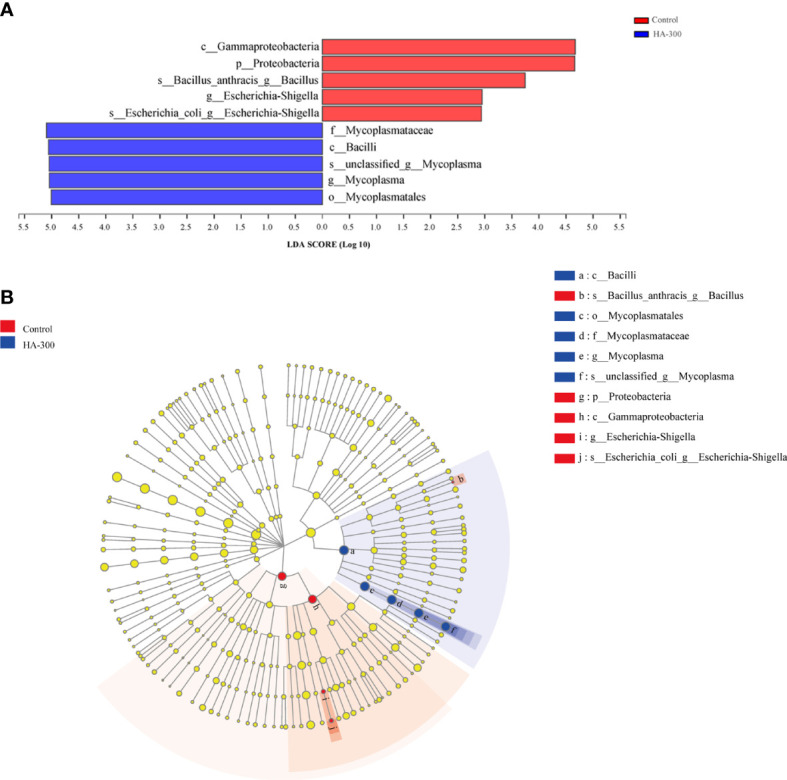
Linear discriminant analysis effect size (LEfSe) analysis of intestinal microflora of largemouth bass. **(A)** Histogram of linear discriminant analysis (LDA) value, with the length representing the LDA score (LDA >2). **(B)** Evolutionary branch diagram, with yellow nodes indicating no significant difference in intestinal microflora, and the red and blue nodes representing the differential microbiota classes that play a significant role in the control and HA-300 groups, respectively.

LEfSe analysis showed that a total of 10 taxa with significant differences between the control and HA-300 groups were found (**Figure 6**). Fish fed diet containing 300 mg kg^-1^ β-glucan significantly increased the relative abundance of *Bacilli* (mainly *Mycoplasmatales*, *Mycoplasmataceae*, *Mycoplasma*) and significantly decreased *Proteobacteria* (mainly *Gammaproteobacteria*, *Escherichia-Shigella*, and *Bacillus anthracis*) (LDA score >2 and *P* < 0.05).

## 4 Discussion

β-glucan have been proven to be a highly efficient stimulator of cellular and humoral branches in mammals and also is a potential stimulant with pronounced immune effects in fish. The effects of dietary β-glucan on growth have been evaluated in different species of aquatic animals, but inconsistent results have been obtained. A study in *L. rohita* showed that 250 and 500 mg kg^-1^ β-glucan significantly enhanced SGR (7). Similarly, Dawood et al. (11) found that 250–1,000 mg kg^-1^ β-glucan supplementation in the feed significantly increased WGR and SGR of red seabream (*Pagrus major*). Conversely, other studies have reported that dietary β-glucan had no significant effect on growth performance (26–29). A study showed that diet supplemented with different levels of β-glucan had no adverse effects on Nile tilapia (*Oreochromis niloticus*) during 10 weeks of feeding (30), which was in accordance with the results of this study. Contradictory results on the influence of β-glucan on growth performance may be due to species, feed composition, breeding environment, or other experimental conditions. Actually, it is generally believed that β-glucan has no direct growth-promoting effect on animals, but affects growth performance by improving immunity. Thus, we further investigated the effect of β-glucan on the immunity of largemouth bass.

The fish immune system is composed of two components, innate and adaptive immunity, in which innate immunity plays a major immune conditioning role. The components of innate immunity are divided into humoral molecules, in which LZM can destroy the cell wall of Gram-positive bacteria and prevent bacterial invasion. In addition, LZM has been recognized as a biomarker of immune defense mechanisms in fish (31, 32). Results of this study showed that LZM activity significantly increased with increasing dietary β-glucan supplementation, suggesting that β-glucan could improve the immunity of largemouth bass. Similar results were also found in other fish species, including the Persian sturgeon (*Acipenser persicus*), Nile tilapia, and hybrid striped bass (*Morone chrysops* × *M. saxatilis*) (33–35). Both *in vivo* and *in vitro* studies revealed that β-glucan significantly improved serum LZM activity in hybrid striped bass (33). Misra et al. (7) reported that serum LZM activity significantly increased after feeding rohu with β-glucan for 28–42 days. The Persian sturgeon fed diets with 200 and 300 mg kg^-1^ β-glucan showed higher LZM activity than those fed 0 and 100 mg kg^-1^ β-glucan (34), which was highly similar to the results of this study. To further investigate the effects of β-glucan on the immunity of largemouth bass, we conducted a challenge experiment with *A. schubertii. A. schubertii* is widely distributed in aquatic environments and also a common pathogen in aquaculture. *A. schubertii* belongs to *Aeromonas mesophilic*, which is one of the most serious bacteria in fish farming (36). Infected fish with *A. schubertii* shows fin rot and hemorrhage on the body surface, mainly causing bacterial septicemia and bacterial enteritis, leading to mass mortality and serious economic losses (37). A study in largemouth bass has confirmed that fish (~15 g) infected with *A. schubertii* (5 × 10^6^ CFU ml^-1^) showed slight hyperemia and hemorrhage of the anus and caudal fin and severe hyperemia of the liver with white nodules (3). The injection concentration of this experiment is higher than the above study mainly due to the fish weight specification. In the present study, all fish fed diets without β-glucan supplementation died after the 12-h challenge, but fish in the β-glucan supplementation groups survived after the 72-h challenge, suggesting that β-glucan could improve the resistance of largemouth basses against *A. schubertii*. Similar results were also found in other studies. A study on Nile tilapia reported that β-glucan can enhance the antioxidant and immune responses to avoid *A. hydrophila* (a branch of *A. schubertii*) infection (38). Meshram et al. (39) reported that a diet supplemented with 1 g kg^-1^ β-glucan enhanced immunity and resistance against *A. hydrophila* in freshwater prawn (*Macrobrachium rosenbergii*).

The transcriptome results showed that a total of 1,245 DEGs were obtained, fish fed a diet with 300 mg kg^-1^ β-glucan showed 449 significantly upregulated DEGs and 796 significantly downregulated DEGs compared with the control group. Meanwhile, 109 immune-related DEGs was screened, with 47 significantly upregulated immune-related DEGs enriched into 12 immune pathways, among which the chemokine signaling pathway and NOD-like receptor signaling pathway have important physiological functions in fish. The chemokine signaling pathway regulates leukocyte migration and plays an important role in nonspecific and specific immune responses in fish (40). Fish chemokines are involved in almost all functions of lymphocytes and can recruit and activate leukocytes to act at the infected site and participate in the immune response (41). In this study, diet supplementation of β-glucan significantly upregulated the expression levels of chemokine family genes, including chemokine XC receptor 1 (*xcr1*), C-X-C chemokine receptor type 1 (*cxcr1*), C-C motif chemokine 7 (*ccl7*), and C-C motif chemokine 5 (*ccl5*), suggesting that the chemokine signaling pathway plays an important role in β-glucan-mediated immune enhancement. In addition, β-glucan significantly upregulated the expression level of protein (Cdc42/Rac)-activated kinase 1 (*pak1*), which is a serine-threonine kinase and plays an important role in regulating key nodes of cellular function and angiogenesis (42). Recently, Ren et al. (43) reported that the activity of LZM significantly decreased in coelomic fluid of sea cucumber (*Apostichopus japonicus*) after inhibition expression of *pak1*. Therefore, the enhanced LZM activity of largemouth bass fed a diet with β-glucan supplementation may be related to the upregulated expression level of *pak1*. Another β-glucan-related immune pathway identified in this study is NOD-like receptor signaling pathway, which plays a key role in pathogen recognition and nonspecific immune responses and lead to the initiation of antimicrobial and antiviral immune responses. Nucleotide-binding and oligomerization domain (NOD)-like receptors (NLRs) are important pathogen recognition receptors in this pathway and play an important role in the nonspecific immune response of teleost fish (44). Studies have confirmed that β-glucan activates NOD-like receptor signaling pathway through NLRs (45). The Nile tilapia *nlrc3* gene was expressed in tissues as NOD1 and NOD2, and the expression was significantly upregulated after *Streptococcus agalactiae* infection (46). Significantly upregulated NLR family genes were also observed in this study, such as NLR family CARD domain-containing protein 3 (*nlrc3*), indicating that the NOD-like receptor signaling pathway is another important pathway in which β-glucan modulates immunity. Furthermore, we also found 62 significantly downregulated immune-related DEGs enriched into 24 immune pathways, among which intestinal immune network for IgA production, cytosolic DNA-sensing pathway, and C-type lectin receptor signaling pathway have important physiological functions in fish. The intestinal immune network for IgA production protects the host from pathogen invasion (47). DNA-directed RNA polymerase (*polr*) complexes play an important role in the immune-related cytosolic DNA-sensing pathway (48). In addition, studies had shown that the C-type lectin receptor signaling pathway stimulated by β-glucan can activate the NF-kappa B signaling pathway to produce an inflammatory response (49), while genes regulated in these pathways including nectin-4-like, *il-10*, *il-1β*, TNFAIP3-interacting protein 1-like (*tnip1*-like), polymerase (RNA) III polypeptide D (*polr3d*), polymerase (RNA) III polypeptide C (*polr3c*), and RNA polymerase II, I and III subunit H (*polr2h*) were significantly downregulated. *Nectin-4* belongs to the family of immunoglobulin-like cell adhesion molecules and causes viral nervous necrosis in grouper (*Epinephelus fuscoguttatus♀× Epinephelus lanceolatus♂*) (50). *Il-10* is an anti-inflammatory factor that inhibits the expression of pro-inflammatory factor *il-1β* and plays a central role in regulating inflammatory responses (51). Studies had shown that β-glucan could downregulate the expression of *il-1β* in common carp (*Cyprinus carpio*), which was consistent with the results of this study (52). *Tnip1* is an inflammation-related gene with multiple roles and expression in multiple cell types (53), while genes of *polr3d*, *polr3c*, and *polr2h* associated with pathogens (54). Overall, transcriptome results of this study demonstrated that diet supplementation of β-glucan upregulated the chemokine signaling pathway and NOD-like receptor signaling pathway (including genes of *xcr1*, *cxcr1*, *ccl7*, *ccl5*, *pak1*, and *nlrc3*) and downregulated the intestinal immune network for IgA production, cytosolic DNA-sensing pathway, and C-type lectin receptor signaling pathway (including genes of *nectin-4*-like, *il-10*, *il-1β*, *tnip1*-like, *polr3d*, *polr3c*, and *polr2h*) to reduce inflammation and enhance immunity in largemouth bass.

Intestinal microorganisms form a complex microbial community in the gastrointestinal tract of animals, which plays an important role in immune function and prevention of pathogen invasion, and are an important indicator for evaluating fish health status. In this study, *Fusobacteria*, *Firmicutes*, and *Proteobacteria* were the dominant bacteria in largemouth bass at the phylum level, which was consistent with results of other studies in largemouth bass (55–58). Some pathogenic bacteria in the intestine can cause reduced immunity in fish, such as *Escherichia-Shigella* and *Escherichia coli* in *Proteobacteria* and *B. anthracis*. Studies have shown that increased relative abundance of *Proteobacteria* is a marker of community instability and intestinal inflammatory response (57–59). *E. coli*, including the closely related genus *Shigella*, is a representative bacterium of highly pathogenic bacterium *Escherichia* (60). *Escherichia-Shigella* is a pathogen that causes intestinal disease, which is positively correlated with intestinal inflammation and negatively correlated with growth performance in fish (61, 62). *B. anthracis* belongs to the genus *Bacillus*, which is a well-known pathogen that can infect skin, lungs, and intestines and cause severe damage to tissues and organs (63). Brown et al. (64) reported that the abundance of potential pathogenic *Vibrio* appeared to be inversely correlated with *Mycoplasma*, suggesting that *Mycoplasma* may be potentially beneficial to rainbow trout. In this study, LEfSe analysis showed that fish fed a diet containing 300 mg kg^-1^ β-glucan significantly decreased the abundance of *Proteobacteria* (mainly *Escherichia-Shigella* and *E. coli*) and *B. anthracis*, suggesting that β-glucan can maintain intestinal health by reducing harmful bacteria.

## 5 Conclusion

In conclusion, long-term oral administration of β-glucan (<300 mg kg^-1^) had no adverse effects on largemouth bass. In addition, 300 mg kg^-1^ β-glucan supplementation stimulated the nonspecific immune system of largemouth bass and improved resistance against *A. schubertii*. Transcriptome analysis revealed that fish fed a diet supplemented with β-glucan significantly upregulated the chemokine signaling pathway and NOD-like receptor signaling pathway and downregulated the intestinal immune network for IgA production, cytosolic DNA-sensing pathway, and C-type lectin receptor signaling pathway. In addition, β-glucan can maintain intestinal health by decreasing harmful bacteria *Proteobacteria* (mainly *Escherichia-Shigella* and *E. coli*) and *B. anthracis* in largemouth bass intestine.

## Data availability statement

The datasets presented in this study can be found in online repositories. The names of the repository/repositories and accession number(s) can be found below: https://www.ncbi.nlm.nih.gov/ , SRR21783450, SRR21783451, SRR21783452, SRR21783453, SRR21783454, SRR21783455 https://www.ncbi.nlm.nih.gov/ , SRR21783676, SRR21783677, SRR21783678, SRR21783679, SRR21783680, SRR21783681.

## Ethics statement

The study was performed in strict accordance with the Laboratory Animal Welfare Guidelines of China (Decree No. 2 of Ministry of Science and Technology, issued in 1988).

## Author contributions

MG performed formal analysis, investigation and writing original draft. YZ and BS performed conceptualization, designed experiment, funding acquisition, supervision and writing review and editing. NL performed data curation and project administration. ZD and JX performed methodology and validation. LC and BW performed data curation and validation. LL and LR performed project administration. All authors contributed to the article and approved the submitted version.
