# Supplementary material for: New insights into β-glucan-enhanced immunity in largemouth bass Micropterus salmoides by transcriptome and intestinal microbial composition
Source: Front Immunol. 2022 Dec 14;13:1086103. doi: 10.3389/fimmu.2022.1086103 (PMC9794605; doi:10.3389/fimmu.2022.1086103)
Supplement: Supplementary file 1 [file DataSheet_1.docx]

Table S1. Formulation and chemical composition of diets (dry matter basis)

| Ingredients, g kg^-1^ | Control | LA-100 | MA-200 | HA-300 |
| --- | --- | --- | --- | --- |
| Peru fish meal | 200 | 200 | 200 | 200 |
| Domestic fish meal | 180 | 180 | 180 | 180 |
| Poultry by-product meal | 80 | 80 | 80 | 80 |
| Soybean meal | 110 | 110 | 110 | 110 |
| Soy protein concentrate | 120 | 120 | 120 | 120 |
| Wheat gluten | 70 | 70 | 70 | 70 |
| Wheat flour | 86.9 | 86.4 | 85.9 | 85.4 |
| Tapioca | 30 | 30 | 30 | 30 |
| Fish oil | 50 | 50 | 50 | 50 |
| Soybean oil | 50 | 50 | 50 | 50 |
| Vita & Mineral premix ^1^ | 10 | 10 | 10 | 10 |
| Ca(H_2_PO_4_)_2_ | 10 | 10 | 10 | 10 |
| Choline chloride | 0.30 | 0.30 | 0.30 | 0.30 |
| *DL*-Met | 1.50 | 1.50 | 1.50 | 1.50 |
| Anti-mold | 0.50 | 0.50 | 0.50 | 0.50 |
| Anti-oxidant | 0.35 | 0.35 | 0.35 | 0.35 |
| Yttrium | 0.50 | 0.50 | 0.50 | 0.50 |
| β-glucan ^2^ | 0.00 | 0.50 | 1.00 | 1.50 |
| Total | 1000 | 1000 | 1000 | 1000 |
| Analyzed content, g kg^-1^ |  |  |  |  |
| Moisture | 78 | 66 | 73 | 64 |
| Crude protein | 523 | 534 | 534 | 537 |
| Total fat | 156 | 156 | 158 | 154 |
| Gross energy, MJ kg^-1^ | 21.8 | 21.6 | 21.9 | 21.6 |

^1^ The vita & mineral premix was described in detail previously (Zhang et al., 2022).

^2^ β-glucan was provided by Kemin AquaScience (Zhuhai, China).


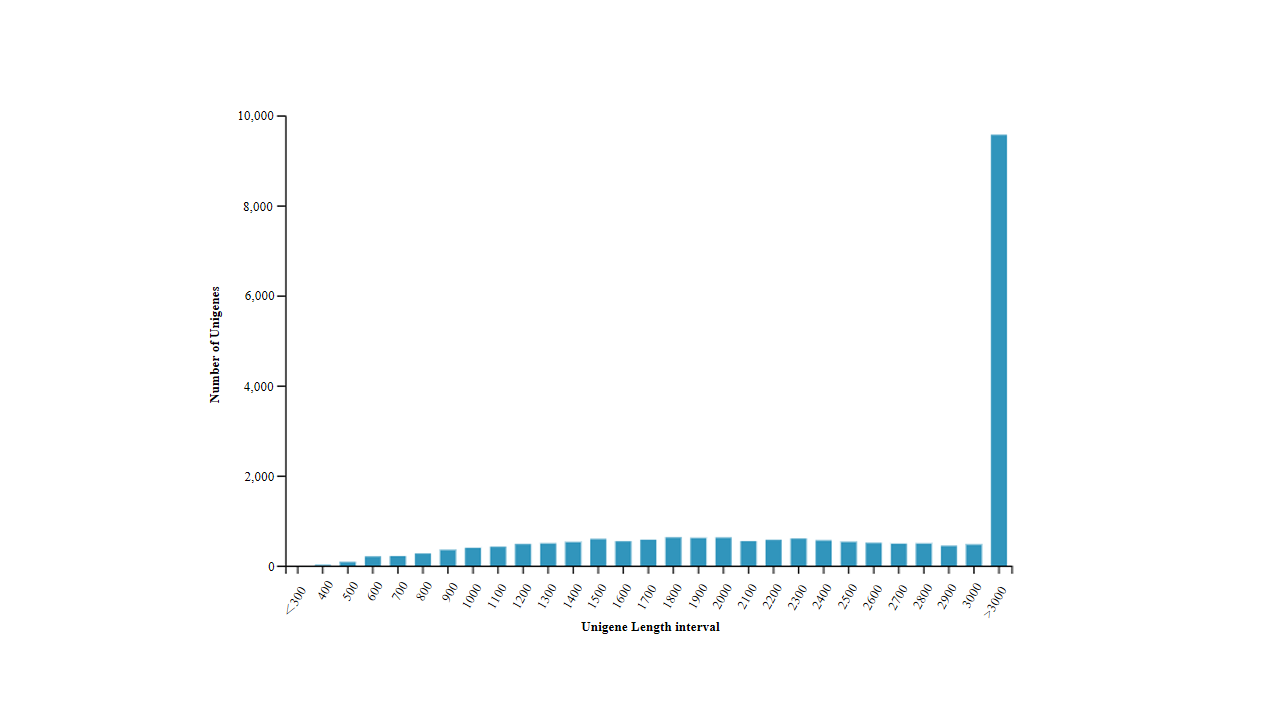


**Fig. S1.** Overview of transcriptome sequence length distributions of largemouth bass.


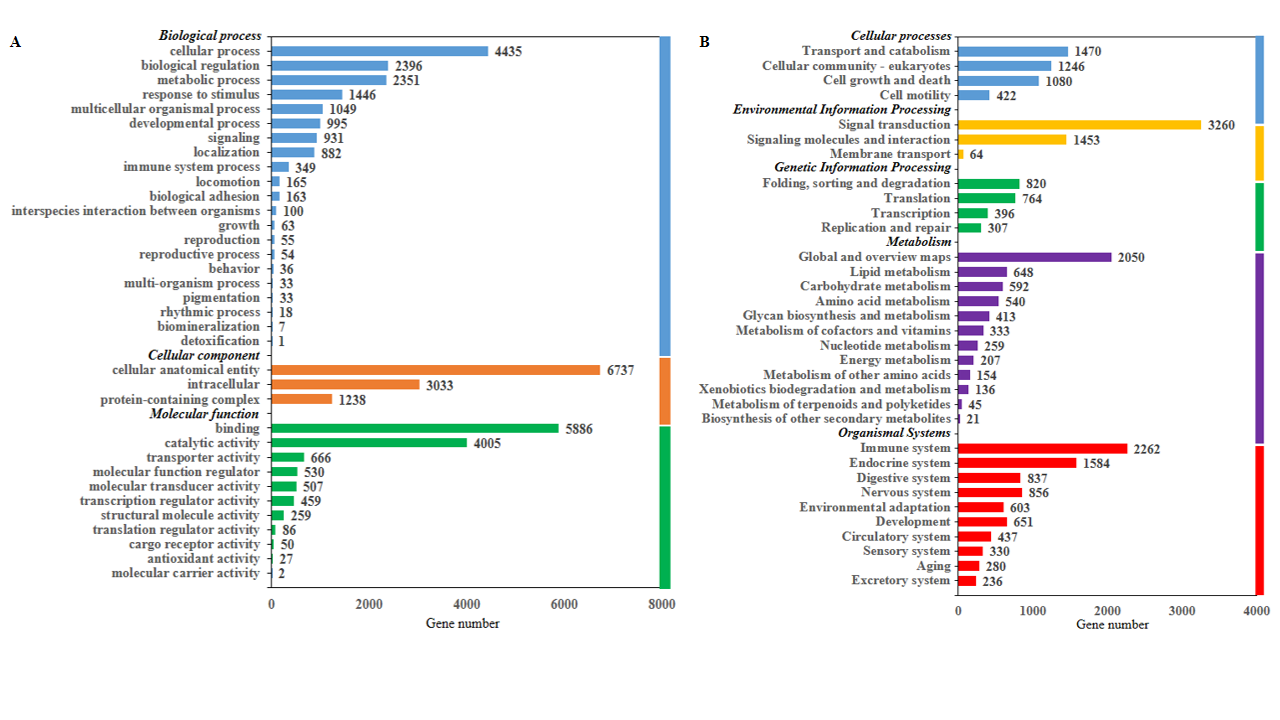


**Fig. S2.** GO annotations (**A**) and KEGG classification (**B**) of all unigenes in live transcriptome of largemouth bass.

**Table S2.** Immune-related DEGs (|log_2_fold change| ≥ 1, *q*-value ≤ 0.05) in liver transcriptome of largemouth bass

| Gene description (name) | Log_2_ (fold change) | *q*-vaule |
| --- | --- | --- |
| ***Up-regulated*** |  |  |
| DBF4-type zinc finger-containing protein 2-like (*zdbf2*-like) | 5.19 | 3.88e-5 |
| calreticulin-like (*calr*-like) | 4.63 | 1.06e-3 |
| chemokine XC receptor 1 (*xcr1*) | 3.81 | 0.01 |
| protein (Cdc42/Rac)-activated kinase 1 (*pak1*) | 3.59 | 0.04 |
| NLR family CARD domain-containing protein 3-like (*nlrc3*) | 3.54 | 3.68e-3 |
| C-X-C chemokine receptor type 1 (*cxcr1*) | 2.95 | 1.10e-4 |
| ubiquitin carboxyl-terminal hydrolase CYLD-like (*cyld*-like) | 2.88 | 2.33e-3 |
| complement factor H-like (*cfh*-like) | 2.36 | 3.37e-67 |
| matrix metalloproteinase-18-like (*mmp18*-like) | 2.01 | 0.03 |
| transient receptor potential cation channel subfamily M member 2-like (*trmp2*-like) | 1.98 | 0.01 |
| protein-tyrosine kinase 2-beta-like (*ptk2b*-like) | 1.96 | 0.04 |
| myelin protein P0-like (*mpz*-like) | 1.88 | 0.02 |
| GRB2 related adaptor protein 2b (*grap2b*) | 1.75 | 1.46e-4 |
| KIT proto-oncogene, receptor tyrosine kinase b (*kitb*) | 1.65 | 0.01 |
| interleukin-8-like (*il8*-like) | 1.59 | 0.04 |
| urokinase plasminogen activator surface receptor-like | 1.48 | 0.03 |
| protein NLRC3-like | 1.41 | 0.01 |
| tripartite motif containing 110 (*trim110*) | 1.38 | 6.25e-5 |
| NACHT, LRR and PYD domains-containing protein 12-like (*nlrp12*) | 1.38 | 2.43e-4 |
| ICOS ligand-like (*icosl*-like) | 1.37 | 0.04 |
| C-C motif chemokine 7 (*ccl7*) | 1.34 | 1.96e-194 |
| coagulation factor X-like (*f10*-like) | 1.31 | 0.00 |
| protein S100-B-like | 1.28 | 2.65e-3 |
| voltage-dependent anion-selective channel protein 2-like (*vdac2*-like) | 1.27 | 1.14e-3 |
| fms related receptor tyrosine kinase 3 (*flt3*) | 1.27 | 0.02 |
| leucine rich repeat containing 39 (*lrrc39*) | 1.14 | 6.06e-17 |
| protein jagged-1a-like | 1.11 | 5.41e-4 |
| KIAA1522 ortholog (*kiaa1522*) | 1.11 | 4.15e-4 |
| mitogen-activated protein kinase kinase 7 (*map2k7*) | 1.11 | 0.03 |
| B2 bradykinin receptor-like | 1.09 | 4.25e-10 |
| CMRF35-like molecule 1 (*clm1*) | 1.08 | 4.00e-4 |
| C-C motif chemokine 5-like (*ccl5*) | 1.08 | 1.67e-3 |
| claudin 11a (*cldn11a*) | 1.04 | 0.02 |
| permeability factor 2-like | 1.04 | 2.30e-3 |
| P2X purinoceptor 1-like | 1.01 | 0.02 |
| ***Down-regulated*** |  |  |
| TNFAIP3-interacting protein 1-like (*tnip1*-like) | -4.31 | 4.06e-3 |
| polymeric immunoglobulin receptor-like (*plgr*-like) | -3.69 | 1.71e-3 |
| arrestin β 1 (*arrb1*) | -3.58 | 0.04 |
| arrestin 3b, retinal (X-arrestin) (*arr3b*) | -3.58 | 0.04 |
| septin-2-like (*septin2*-like) | -3.34 | 0.01 |
| protein FAM110A-like | -3.17 | 0.01 |
| synaptogyrin 3a (*syngr3a*) | -3.16 | 0.02 |
| protein disulfide-isomerase A3-like | -2.80 | 4.13e-3 |
| nectin-4-like | -2.80 | 0.05 |
| interleukin 1, beta (*il1β*) | -2.80 | 4.13e-3 |
| interleukin-8 (*il8*) | -2.71 | 6.10e-39 |
| coagulation factor XIII A chain-like (*f13a*-like) | -2.71 | 1.04e-7 |
| interferon regulatory factor 4-like (*irf4*-like) | -2.41 | 3.93e-7 |
| early growth response 2b (*egr2b*) | -2.31 | 0.01 |
| low affinity immunoglobulin gamma Fc region receptor II-c-like | -2.27 | 1.95e-4 |
| tumor necrosis factor receptor superfamily member 13B-like (*tnfrsf13b*-like) | -2.03 | 0.03 |
| proto-oncogene tyrosine-protein kinase Src-like | -1.99 | 0.03 |
| V-set domain-containing T-cell activation inhibitor 1-like (*vtcn1*-like) | -1.99 | 1.94e-5 |
| lysozyme g-like (*lyg*-like) | -1.90 | 0.02 |
| synaptogyrin-1-like (*syngr1*-like) | -1.87 | 0.05 |
| GTP-binding protein Rhes-like (*rhes*-like) | -1.83 | 4.10e-6 |
| interleukin 12 receptor, beta 2a like (*il12rb2l*) | -1.75 | 5.80e-165 |
| ankyrin repeat domain-containing protein 45-like | -1.69 | 0.03 |
| interleukin 10 (*il10*) | -1.67 | 0.03 |
| myosin, light chain 7 (*myl7*) | -1.67 | 0.03 |
| DNA-directed RNA polymerases I and III subunit RPAC1-like (*rpac1*-like) | -1.66 | 3.78e-11 |
| toll-like receptor 9 (*tlr9*) | -1.58 | 3.42e-3 |
| MARCKS-like 1b (*marcksl1b*) | -1.54 | 3.87e-6 |
| heat shock protein 4a (*hspa4a*) | -1.54 | 1.13e-31 |
| transferrin receptor 1b (*tfr1b*) | -1.51 | 9.99e-12 |
| nectin-4-like | -1.49 | 4.44e-3 |
| phospholipid phosphatase 1-like | -1.42 | 2.95e-15 |
| polymerase (RNA) III (DNA directed) polypeptide D (*polr3d*) | -1.38 | 1.41e-12 |
| THADA armadillo repeat containing (*thada*) | -1.33 | 2.45e-14 |
| heat shock 70 kDa protein 1-like (*hspa1l*) | -1.29 | 0.05 |
| tumor necrosis factor receptor superfamily member 10A-like (*tnfrsf10a*-like) | -1.18 | 0.05 |
| tetratricopeptide repeat protein 27-like (*ttc27*-like) | -1.12 | 0.01 |
| claudin-5-like (*cldn5*-like) | -1.11 | 1.26e-6 |
| polymerase (RNA) III (DNA directed) polypeptide C (*polr3c*) | -1.09 | 5.31e-11 |
| DNA-directed RNA polymerases I and III subunit RPAC1-like (*rpac1*-like) | -1.06 | 2.99e-9 |
| nuclear receptor coactivator 7-like (*ncoa7*-like) | -1.06 | 0.04 |
| RNA polymerase II, I and III subunit H (*polr2h*) | -1.03 | 0.01 |
| antithrombin-III-like (*atⅢ*-like) | -1.01 | 0.00 |
| cathepsin L.1 (*ctsl.1*) | -1.01 | 1.10e-6 |


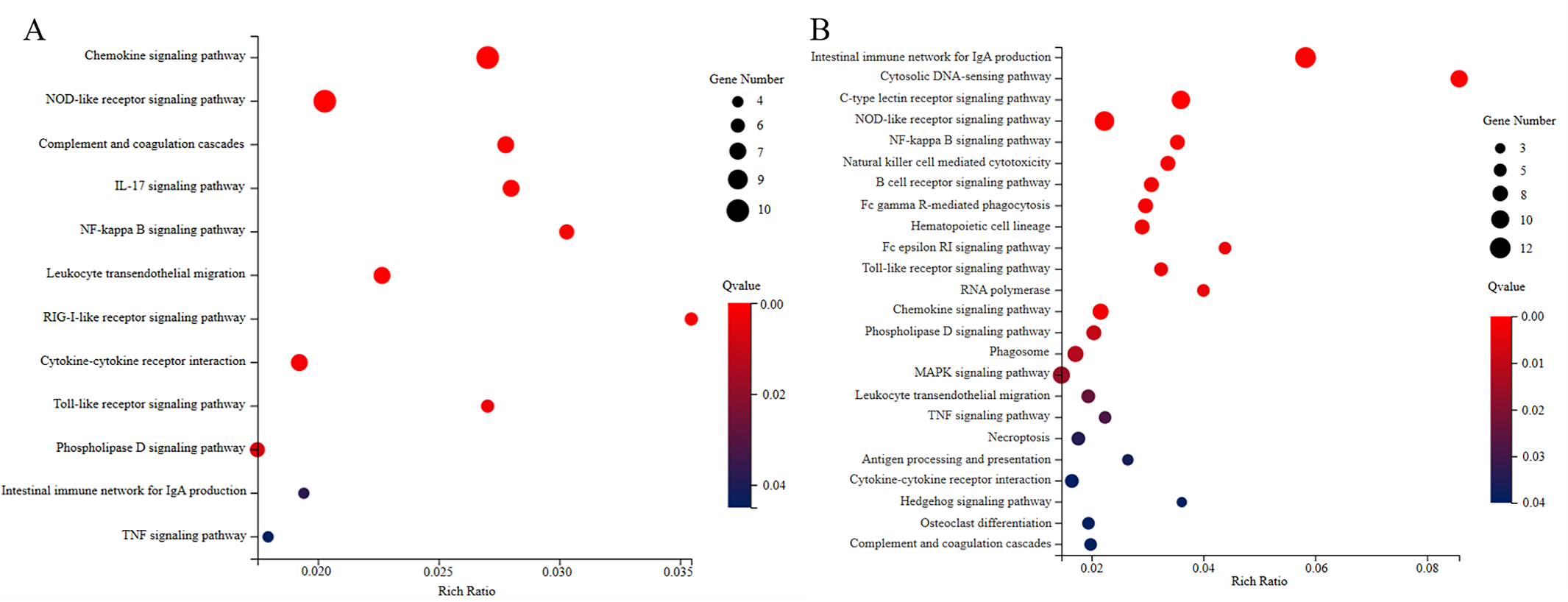
**Fig. S3.** Up-regulated (**A**) and down-regulated (**B**) of immune-related DEGs in liver transcriptome of largemouth bass after KEGG pathway enrichment.

The parameters were calculated as follows:

Weight gain rate (WGR, %) = (final body weight - initial body weight) / initial body weight ×100;

Specific growth rate (SGR, %/d) = [ln (final mean body weight) - ln (initial mean body weight)] / days ×100;

Feed conversion ratio (FCR) = feed intake / (final mean body weight - initial mean body weight);

Hepatosomatic index (HSI, %) = liver weight / body weight ×100;

Viscerosomatic index (VSI, %) = viscera weight / body weight ×100;

Condition factor (CF, 100g/cm^3^) = body weight / body length^3^×100;

Gonadosomatic index (GSI, %) = (gonad weight / body weight) ×100;

Protein retention efficiency (PRE, %) = protein gain / protein intake ×100;

Energy storage ratio (ERE, %) = energy gain / energy intake ×100.

**References:**

Zhang Y, Zhang L, Huang L, Dong Z, Lu Q, Zou Y, Tang F, Zhao S, Storebakken T. Evaluation of conventional or hydrolyzed stickwater from food-grade skipjack tuna by-product in diet for hybrid grouper (*Epinephelus fuscoguttatus♀× Epinephelus lanceolatus♂*). Aquaculture 2022 548: 737714.
